# Synthesis and Biological Evaluation of Phosphate Prodrugs of 4-Phospho-d -erythronohydroxamic Acid, an Inhibitor of 6-Phosphogluconate Dehydrogenase

**DOI:** 10.1002/cmdc.200700040

**Published:** 2007-08-13

**Authors:** Gian Filippo Ruda, Vincent P Alibu, Christos Mitsos, Olivier Bidet, Marcel Kaiser, Reto Brun, Michael P Barrett, Ian H Gilbert

**Affiliations:** 1 Division of Biological Chemistry and Molecular Microbiology, College of Life Sciences, University of Dundee, Sir James Black CentreDundee DD1 5EH, UK, Fax: (+44)1382-386-373; 2 Division of Infection & Immunity, Institute of Biomedical and Life Sciences, University of Glasgow, Glasgow Biomedical Research CentreGlasgow G12 8TA, UK; 3 Welsh School of Pharmacy, Cardiff University, Redwood Building, King Edward VII AvenueCardiff, CF10 3XF, UK; 4 Swiss Tropical InstituteSocinstrasse 57, CH-4002 Basel, Switzerland

**Keywords:** 6-phosphogluconate dehydrogenase, masked phosphate, prodrugs, *Trypanosoma brucei*

## Abstract

We have previously reported the discovery of potent and selective inhibitors of 6-phosphogluconate dehydrogenase, the third enzyme of the phosphate pentose pathway, from *Trypanosoma brucei*, the causative organism of human African trypanosomiasis. These inhibitors were charged phosphate derivatives with restricted capacity to enter cells. Herein, we report the synthesis of five different classes of prodrugs: phosphoramidate; bis-S-acyl thioethyl esters (bis-SATE); bis-pivaloxymethyl (bis-POM); CycloSaligenyl; and phenyl, S-acyl thioethyl mixed phosphate esters (mix-SATE). Prodrugs were studied for stability and activity against the intact parasites. Most prodrugs caused inhibition of the growth of the parasites. The activity of the prodrugs against the parasites appeared to be related to their stability in aqueous buffer.

## Introduction

Human African trypanosomiasis (HAT), also known as sleeping sickness, is a life threatening disease that affects many people in sub-Saharan Africa.^1^ It is caused by the protozoan *Trypanosoma brucei,* of which two subspecies (*T. b. rhodesiense* and *T. b. gambiense*)^2^ are pathogenic to humans. These different subspecies give rise to different clinical symptoms. Three of the four drugs used against this disease were developed more than 50 years ago; the fourth one and the most recent, eflornithine (d,l-α-difluoromethylornithine, DFMO) is active only against *T. b. gambiense*. As a result of increasing resistance and the side effects associated with the available drugs there is an urgent need to develop new treatments to fight this disease.^2^

The bloodstream form of *T. brucei* spp are entirely dependent on glycolysis for production of ATP; thus the parasite is susceptible to inhibition of glycolysis, and some of the enzymes involved in the metabolism of glucose are potential targets for the development of new treatments.^3^ Glucose is also metabolised by the pentose phosphate pathway (PPP), the third enzyme of which, 6-phosphogluconate dehydrogenase (6-PGDH), has been shown to be essential for the viability of *T. brucei*.^4^ The enzyme catalyses the conversion of 6-phosphogluconate (6PG) to ribulose 5-phosphate (Ru5P) with concomitant reduction of one mole of NADP^+^ to NADPH. Inhibition of the enzyme will diminish production of NADPH, thus increasing the parasite’s vulnerability to oxidative stress. Moreover, the levels of ribose 5-phosphate needed for nucleotide biosynthesis will decline and 6PG will accumulate in the cell. 6PG is known to be an inhibitor of 6-phosphoglucose isomerase, the enzyme which converts glucose 6-phosphate to fructose 6-phosphate during glycolysis. Inhibition of this enzyme consequently should lead to more glucose 6-phosphate entering the PPP potentially creating a self-feeding loop with lethal consequences for the parasite.^5^

**Figure 1:**
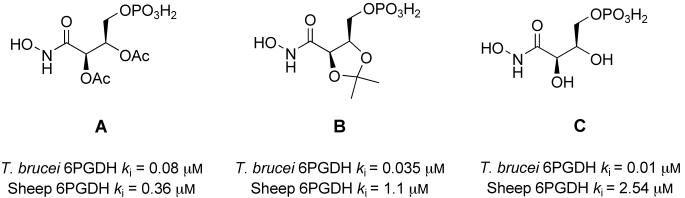
Inhibitors of *T. brucei* 6-PGDH.

Previous work from our group, has identified a series of potent and selective inhibitors of *T. brucei* 6-PGDH^6^,^7^ (Figure 1). Unfortunately these compounds were inactive in vitro against the intact parasite probably because of poor uptake into the parasites. The IC_50_ values of these compounds against *T. b. rhodesiense* were 229 μm, >332 μm, >389 μm for **A**, **B**, and **C** respectively. Low cellular penetration is found with drugs bearing phosphate or phosphonate groups, because of these groups being deprotonated at physiological pH. An increasing number of phosphate esters of pharmaceutical interest (mainly antiviral agents and signaling regulators) has encouraged the advancement of the prodrug approach for the delivery of such compounds into the target cells.^8^,^9^ Several kinds of phosphate masking group have been developed.^8^,^10^–^13^ Different mechanisms then operate to release the parent drug inside the cell. These range from simple chemical hydrolysis^12^ to a multienzymatic cleavage of the prodrugs by the action of several enzymes, mainly esterases.^10^,^13^–^15^

In this paper we discuss the conversion of the 6-PGDH inhibitor **B** into prodrugs to increase its activity against the *T. brucei* by enhancing uptake by passive permeation across the plasma membrane. Five different phosphate-masking groups (phosphoramidate, bis-*S-*acyl thioethyl esters, *bis*-pivaloxymethyl, CycloSaligenyl and phenyl, *S*-acyl thioethyl mixed phosphate esters) have been produced.

The synthesised prodrugs were then evaluated for activity against the parasite *T. brucei brucei*. Their stability was also studied in phosphate-buffered saline (PBS) at 37°C.

## Results and Discussion

### Chemistry

**scheme 1:**
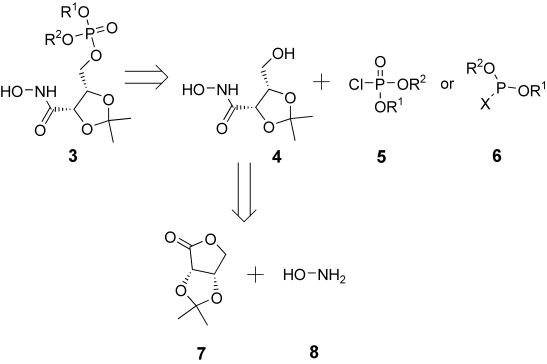
Retrosynthetic analysis for the target prodrugs.

The retrosynthetic analysis of the target compounds is shown in Scheme 1. The synthesis of all five types of masked phosphate can be obtained from the same intermediate: the 2,3-*O*-isopropylidene erythrono hydroxamic acid **4**, which can be coupled with different chloro phosphate diesters **5** or with phosphine-like derivatives **6**.

The acidity of the hydroxamic group was found to be a problem in the synthesis of prodrugs of the 4-phosphate of the erythrono hydroxamic acid **1**. To limit the possible side reactions, a suitable protecting group for the hydroxamic acid was studied. Indeed, attempts at phosphorylating the compound when the hydroxamate was unprotected were unsuccessful and led to complex mixtures.^7^

Our first synthesis of the lead **B** was achieved by opening the 2,3-isopropylidene-d-erythronolactone **7** with *O*-benzyl hydroxylamine followed by phosphorylation with tribenzylphosphite and final cleavage of the benzyl groups by hydrogenolysis.^7^ Unfortunately the benzyl protecting group was too stable to be used in the synthesis of prodrugs, and could not be removed in the presence of the masked phosphate groups. Therefore various other protecting groups for the hydroxamate were investigated. Attempts with *tert*-butyldimethylsilyl (TBDMS), trityl, THP, and polymer-supported benzyl were not successful; instability, low yields, or difficulties monitoring the reaction (in the case of PS-benzyl) were the main problems encountered.

**scheme 2:**
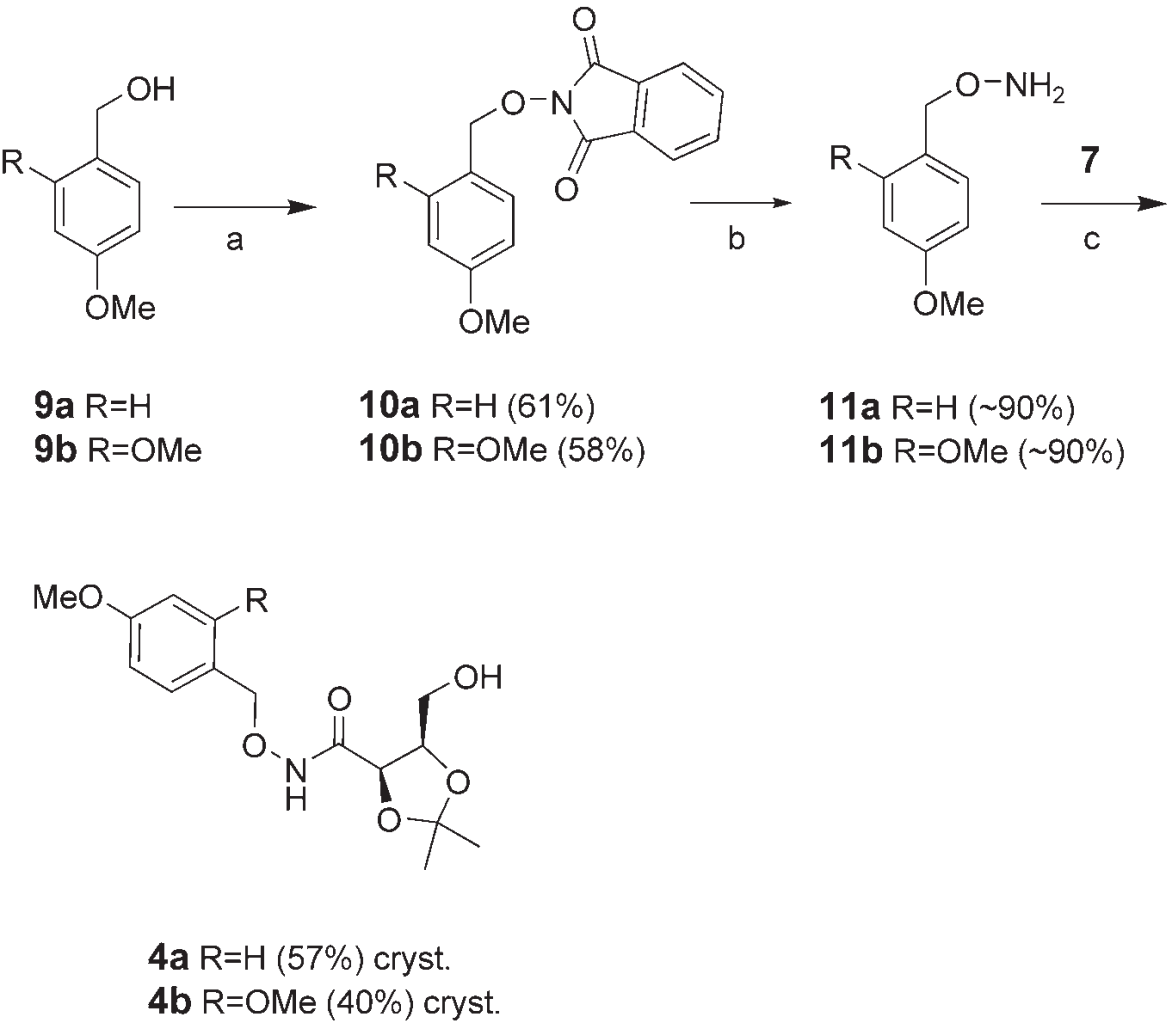
a) *N*-hydroxyphthalimide, DIAD, PPh_3_, THF, RT 48 h; b) CH_3_NHNH_2_, EtOH, reflux, 1 h; c) AlMe_3_, DCM.

In our search for an alternative protective groups we found that *O*-2,4-dimethoxybenzyl and *O*-4-methoxybenzyl hydroxylamines were successfully used by Barlaam et al. for the synthesis of hydroxamic acids.^16^ These protected hydroxylamines can be readily prepared from the 4-methoxybenzyl alcohol **9a** and the 1,4-dimethoxy benzyl alcohol **9b** by Mitsunobu reaction with *N*-hydroxyl phthalimide, followed by removal of the phthalimide protecting group with *N*-methyl hydrazine, Scheme 2.

1,2-Isopropylidene-d-erythronolactone was opened by transamidation with the two protected hydroxylamines^17^ **11a** or **11b** (Scheme 2), in the presence of trimethyl aluminium. The corresponding protected hydroxamic acids **4a** and **4b** were obtained in good yield after recrystallization.

**scheme 3:**
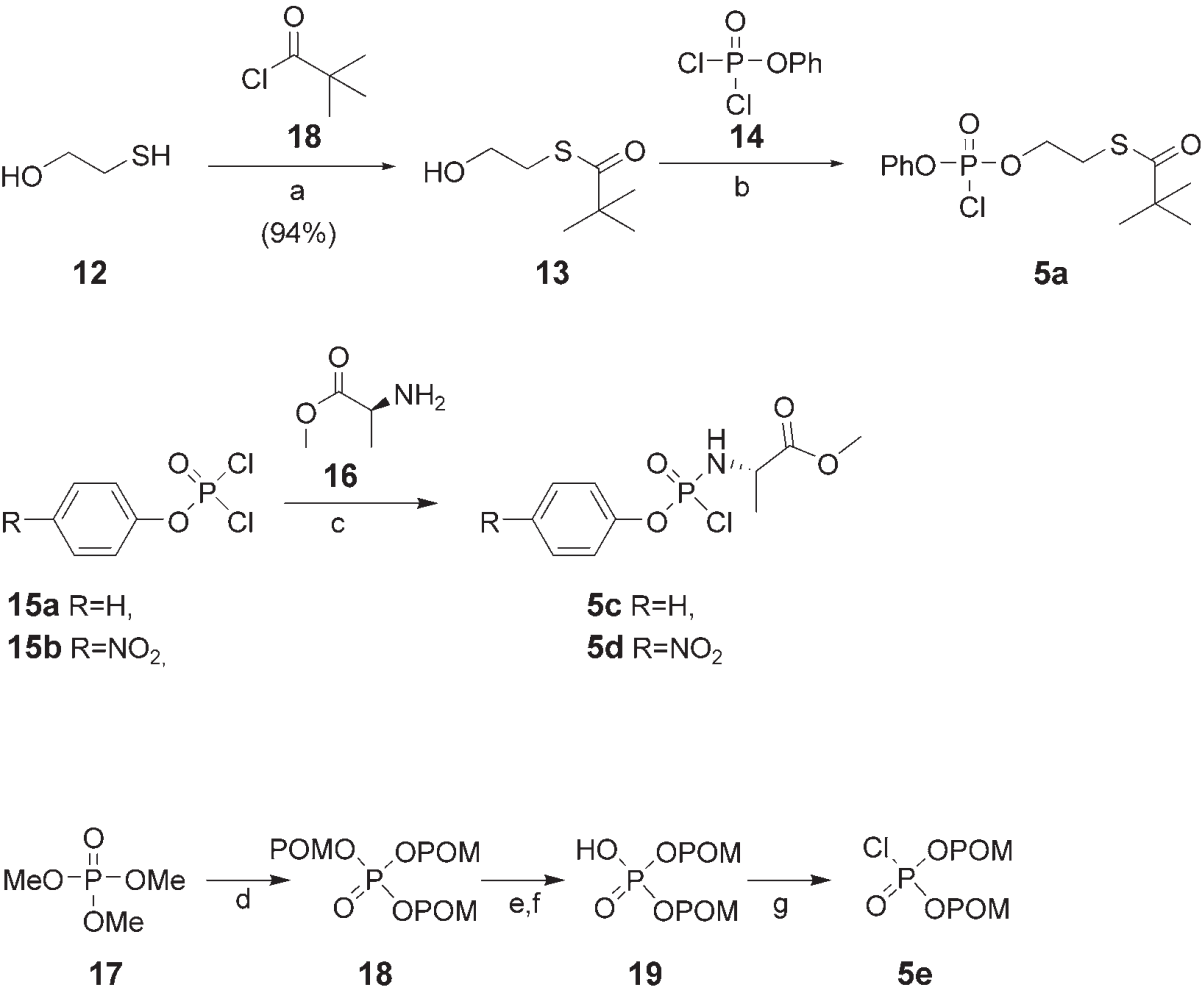
a) TEA, DCM, −78°C; b) TEA, THF, −78°C to RT; c) TEA, DCM RT; d) Chloromethyl pivalate, NaI, CH_3_CN reflux 3 days; e) piperidine RT 12 h; f) Dowex H^+^, water RT 10 h; g) Oxalyl chloride, DMF, RT 2 h.

Phosphoramidates, mix-SATE, and bis-POM precursors for prodrug synthesis were prepared from P^V^ precursors (Scheme 3). The *S*-acyl 2-thioethyl derivative **5a** was obtained in three steps starting from thioethanol **12**, selectively derivatizing with pivaloyl chloride at low temperature.^18^ The *S*-pivaloyl alcohol **13** was then reacted with phenyl dichlorophosphate affording **5a**. The phosphoramidates **5c** and **5d** were synthesised from l-alanine methyl-ester **16**, phenyl dichlorophosphate **15a**, and *p*-nitrophenyl dichlorophosphate **15b** respectively as reported by McGuigan and co-workers.^19^

The *bis*-pivaloxymethyl intermediate **5e** was prepared following the procedure reported by Cole and co-workers^20^ from trimethyl phosphate **17** by *trans*-esterification with chloromethyl pivalate, and hydrolysis to the diester **19**. The final step was the conversion into the chlorophosphate with oxalyl chloride and catalytic dimethyl formamide.

**scheme 4:**
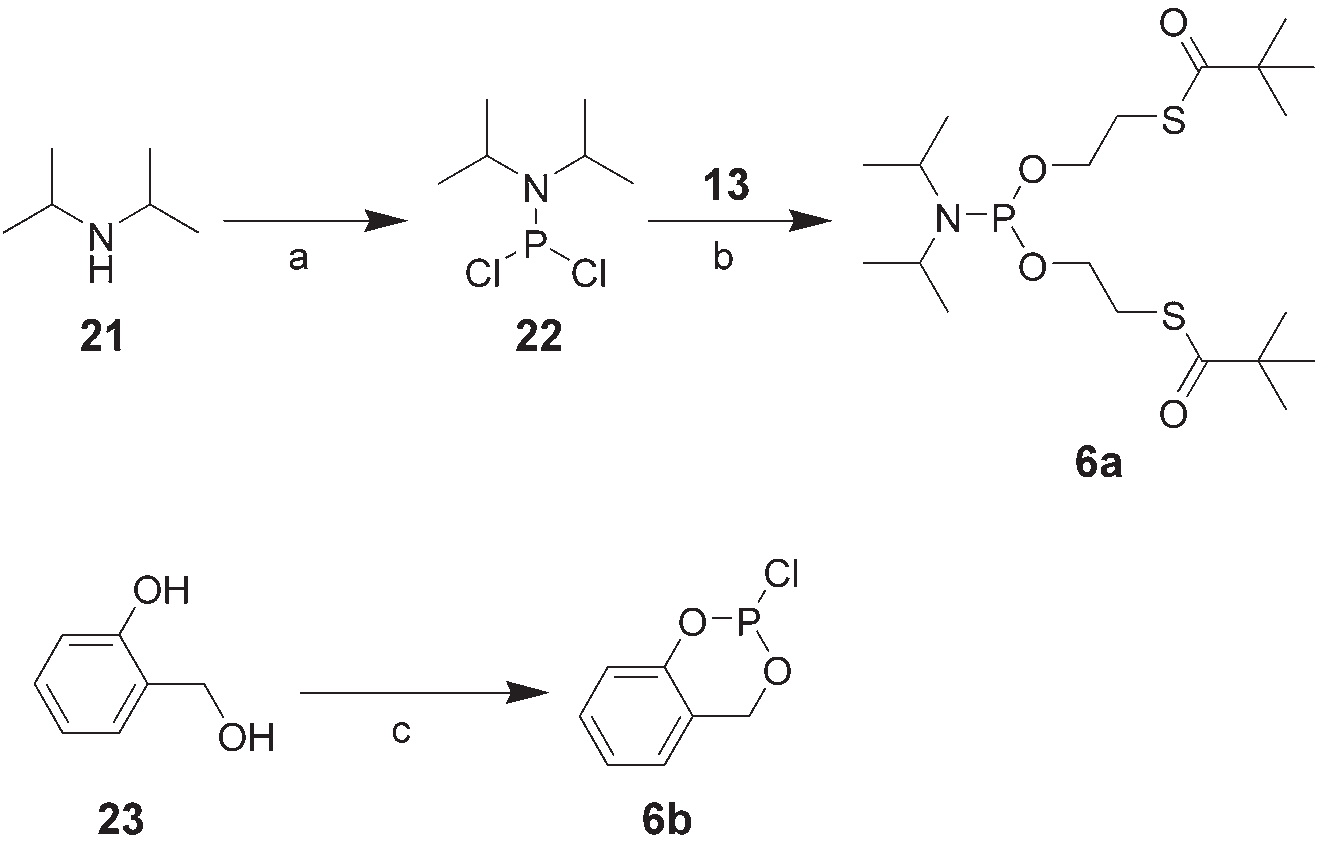
a) PCl_3_, THF, −78°C; b) 13, TEA, THF, −78°C to RT; c) PCl_3_. TEA, Et_2_O, 0°C.

The cycloSal and the bis-*S*-acyl thioethyl esters were prepared using P^III^ chemistry (Scheme 4). The bis-SATE derivative **6a** was obtained by displacement of the chlorides of 1,1-dichloro-*N*,*N*-diisopropylphosphinamine **22** with *S*-pivaloyl ethanol **13**. In a similar method 1-hydroxy benzyl alcohol (salicylic alcohol) **23** was reacted with phosphorous trichloride to afford the corresponding chloro phosphine **6b**.

**scheme 5:**
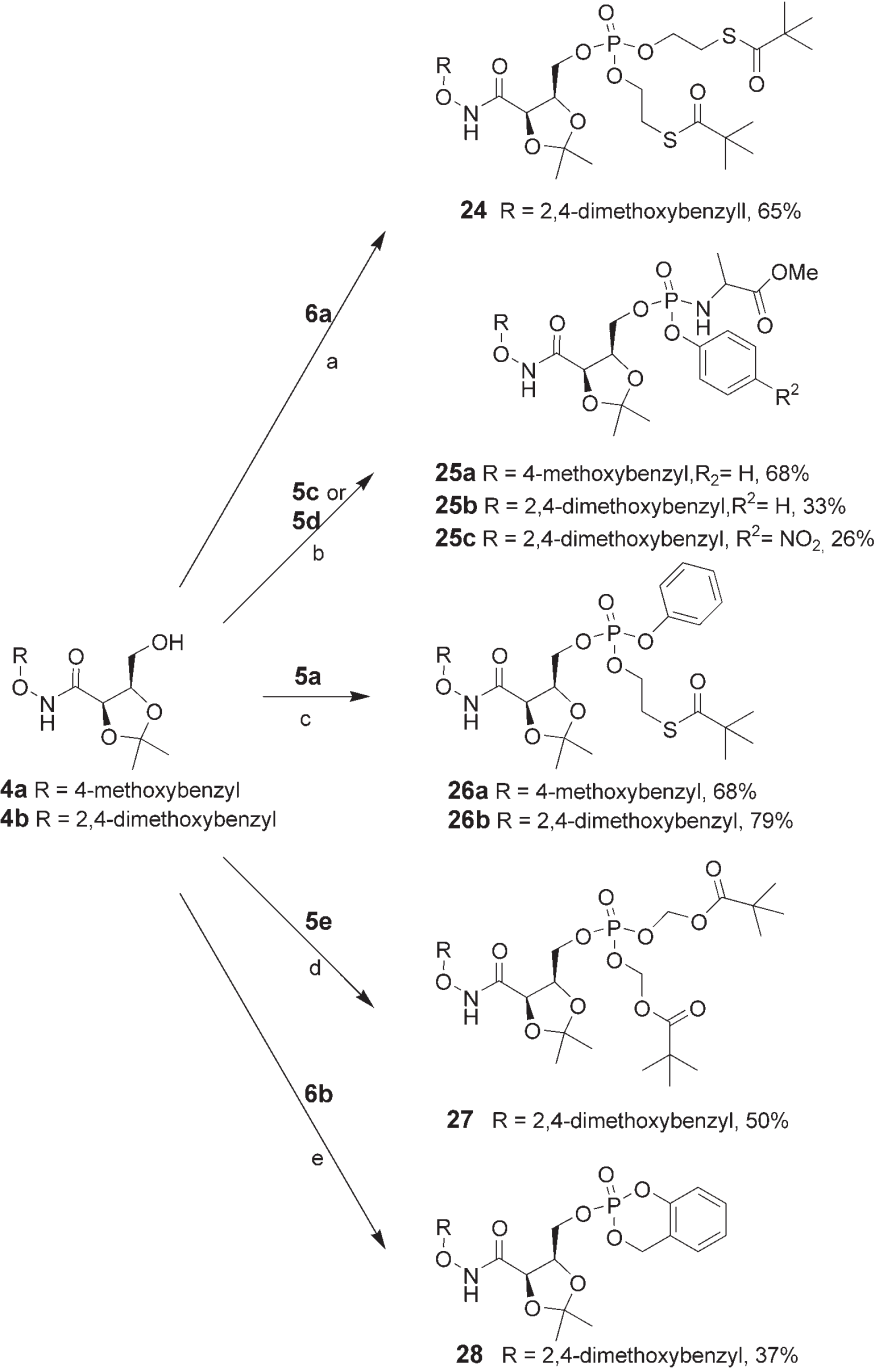
a) 1) Tetrazole, THF, RT, 1 h, 2) *t*BuOOH, −78°C to RT; b) NMI, DCM, −78°C; c) NMI, DCM, −78°C to RT; d) TEA, DCM, −78°C to RT 2 h; e) 1) DIPEA, CH_3_CN, 2) *t*BuOOH, −78°C to RT.

All the intermediates **5a**–**c** and **6a**–**b** were coupled either with the protected hydroxamic derivatives **4a** or **4b** (Scheme 5). The two phosphines **6a**–**b** were reacted with tetrazole first and then with *tert*-butyl hydroperoxide to oxidize the P^III^ to P^V^. The chlorophosphates **5a**–**c** reacted with *N*-methyl imidazole or triethylamine or DIPEA; following the procedures suggested in the literature.

**scheme 6:**
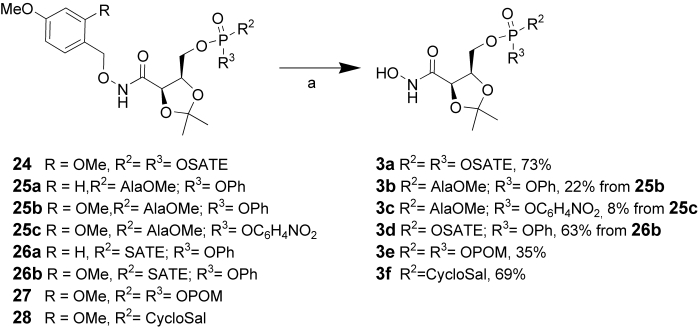
a) TFA, DCM 15 min.

As a final step, the cleavage of the dimethoxybenzyl group was achieved with 1–5% TFA in DCM (Scheme 6). Using these very mild conditions, it was possible to obtain the target prodrugs **3a**–**f** with moderate to good yields. This marks the development of chemistry to cleave a protecting group subsequent to the masked phosphate (prodrug moiety) being introduced into the molecule.

Attempts at cleavage of the monomethoxy benzyl group were not successful, as the higher stability of this required more acidic conditions and longer reaction time (5% TFA in DCM); under these conditions, decomposition of the starting prodrug was observed. An alternate method for removing a 4-methoxybenzyl group is with oxidative conditions (for example, DDQ). However, in our case, DDQ was not able to remove the 4-methoxybenzyl group and, consequently this protecting group was not further investigated.

### Stability studies

The stability of the six prodrugs synthesised was evaluated in phosphate buffer saline at 37°C by LC–MS and ^31^P NMR spectroscopy. The prodrugs were dissolved in buffer, (DMSO was also added in case of poor aqueous solubility), incubated, and analysed hourly until total decomposition was observed.

**Figure 2:**
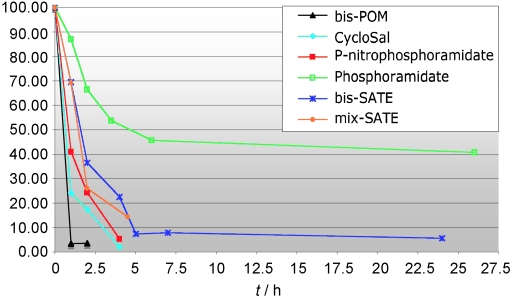
Decomposition curves for the six prodrugs synthesised.

In the LC–MS experiment the disappearance of the molecular ion for the starting prodrug was observed and the declining intensity of the ion current peak was plotted against time, producing the decomposition curve. The half-life of the prodrugs was determined as the time when the intensity of the starting peak was fallen to half of the starting value, Figure 2 and Table 1.

**Table 1:** Half-lives in PBS at 37°C of the prodrugs of the 4-phospho hydroxamic acid. *R*_t_ retention time for the prodrug.

Compd.	Prodrug	*R*_t_ [min]	Half-life [h]
**3b**	Phosphoramidate	5.9	5
**3d**	Mixed-SATE	6.9	2.14
**3a**	Bis-SATE	7.0	1.6
**3c**	P-nitro phosphoramidate	6.0	0.85
**3f**	CycloSal	5.3	0.63
**3e**	Bis-POM	6.7	0.53

In the case of the *p*-nitro phosphoramidate **3c** it was also possible to detect one of the by-products produced by the decomposition of the prodrug in phosphate buffer. This was the corresponding phosphoramidate with the loss of the *p*-nitro phenyl group. Such results could be explained by the effect of the nitro group, which stabilizes the negative charge in the phenolate anion, thus making the hydrolysis of the group easier and therefore this phosphoramidate prodrug has a shorter half-life compared to the corresponding phosphoramidate with the simple phenyl ester **3b**.

Unfortunately in the other cases, although some new peaks, with lower molecular ions compared to the parent prodrugs were identified, a conclusive structure could not be attributed to them and further analysis is undergoing.

**Figure 3:**
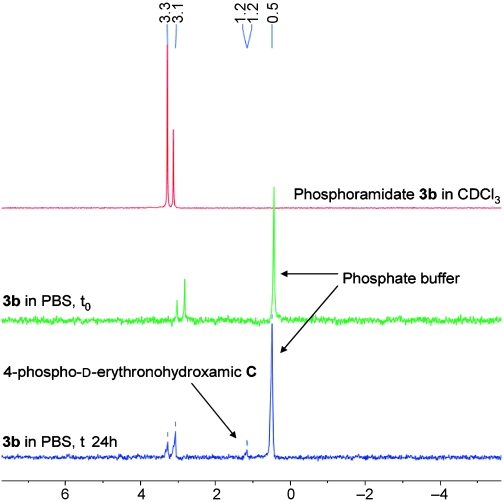
^31^P NMR of the phosphoramidate. Top line pure phosphoramidate in CDCl_3_. Middle line phosphoramidate in PBS at t_0_. Bottom line phosphoramidate in PBS after 24 h at 37°C. The main peak at 0.2 ppm is due to the phosphate buffer.

^31^P NMR spectroscopy also showed the decomposition of the prodrug by the disappearance of the signal for the phosphate prodrugs with time courses comparable with those found by LC–MS. Figure 3 shows the ^31^P NMR spectra for the case of the phosphoramidate **3b**. The two peaks at *δ*=3.3 and 3.1 ppm for the chiral phosphorous of the starting phosphoramidate **3b** are still present after 24 h incubation at 37°C (bottom spectrum) and only one new little peak at *δ*=1.2 ppm is detected probably due to the 4-phospho-d-erythronohydroxamic acid C^7^ (the main peak at 0.5 ppm is due to the phosphate buffer).

### Biological evualuation

The prodrugs **3a**–**f** and some of the protected hydroxamic intermediates were assayed for in vitro activity against *T. b. brucei* (Bs427) and in a counter screen for cytotoxicity against a mammalian cell line (HEK 293T). The IC_50_ values are presented Table 2. The compounds showed activity against the parasite. Whilst further work is required to prove that the killing is by inhibition of 6-PGDH, this result could indicate that the compounds are now able to permeate the cell-membrane, be converted from the prodrug to the active hydroxamate, and then kill the parasite by inhibiting 6-PGDH.

**Table 2:** IC_50_ values for the synthesised prodrug and some intermediates against *T. b. brucei* (Lister 427), and human embryonic kidney cells (HEK 293T) IC_50_ (μm).

Compd.	Structure	Bs427	HEK 293T
**3a**		4.23	NA
**3b**		6.95	NA
**3c**		43.8	NA
**3d**		1.73	NA
**3e**		20.23	NA
**3f**		NA	NA
**24**		25	NA
**26a**		NA	NA
**26b**		NA	NA
**27**		39.11	NA
**28**		NA	NA

NA, not active at highest concentration tested: for Bs427, 100 μm and for HEK 293T, 200 μm.

Compounds **3d**, **3a**, and **3b** showed the highest activity against *T. b. brucei* in decreasing order of activity. Compounds **3e** and **3c** had moderate activities whereas **3f** showed no trypanocidal activity even at 100 μm. There seemed to be a correlation between stability of the compounds in aqueous buffer and in vitro activity.

Interestingly some of the masked hydroxamate analogues (**24** and **27**) also showed improved activity on *T. brucei* strains, which could indicate cleavage of the dimethoxybenzyl moiety under cellular conditions.

Finally none of the compounds tested showed appreciable cytotoxicity against the mammalian cell line HEK293T; indicating good selectivity against trypanosomes. This would be predicted by the selectivities observed for compounds **A**–**C**, which were very selective for the parasite enzyme over the corresponding mammalian one.

## Conclusions

We have developed a new procedure for the synthesis of several classes of phosphate prodrugs in the presence of other potentially interfering groups (that is, hydroxamic acid in our case). The use of the 2,4-dimethoxybenzyl protecting group allowed the introduction of the five masked phosphate groups at the penultimate step of the overall synthesis. The cleavage of the hydroxamate protecting group using very mild conditions (1–2% TFA in DCM in 15 min) was compatible with all the masking groups allowing us to achieve chemoselectivity between the alcohol function and the hydroxamic moiety in the total synthesis.

The stability studies showed that some of the prodrugs have relatively short half-lives in aqueous phosphate buffer at 37°C. Comparison of the measured half-lives with those reported by Azéma et al^21^ for a series of enzyme-labile aldolase inhibitors containing masked phosphates and other data reported for both SATE^18^,^22^ and phosphoramidate^23^ nucleosides indicates that the prodrugs reported herein have shorter half-lives than reported for other compounds where these phosphate masking groups are used. This is presumably due to particular features of the structures of the compounds reported herein. It is possible that the hydroxamic acid or one of the other hydroxyl groups promotes hydrolysis.

Although the mechanism of action has yet to be proven, the activity against the parasites correlates with the stability studies, showing that the compounds with the longest half-lives (the phosphoramidate **3b**, the mixed, and the *bis*-SATE **3a** and **3d**) are the most active in vitro, whereas the least stable are the least active, indicating that their trypanotoxicity is limited by the fact that they could decompose prior to entry into the parasite cells. Future work to address issues of stability will be required before these compounds might be considered for antimicrobial use in vivo.

Biological evaluation shows that the prodrug approach has drastically improved the in vitro activity of the parent compound **B**.^7^ This data also provides further (chemical) validation of 6-PGDH as a drug target, although additional work is required to establish that the cellular mode of action of these compounds is indeed specific inhibition of the enzyme.

In conclusion the chemical synthesis of five different classes of phosphate prodrugs has been achieved. All but one of these compounds show trypanotoxicity

## Experimental Section

^1^H NMR, ^13^C NMR, ^31^P NMR, and 2D-NMR spectra were recorded either on a Bruker Avance DPX 300 spectrometer or on a Bruker Avance DPX 500 spectrometer. Chemical shifts (δ) are expressed in ppm. Signal splitting patters are described as singlet (s), broad singlet (bs), doublet (d), triplet (t), quartet (q), multiplet (m), or combination thereof.

Low resolution electrospray (ES) mass spectra were recorded either on a Applied Biosystem Mariner API-TOF Biospectrometry Workstation spectrometer or on a Bruker MicroTof mass spectrometer, run in either positive or negative ion modes, using either methanol, methanol/water (95:5), or water/acetonitrile (1:1) + 0.2% formic acid as the mobile phase. High resolution electrospray measurements were performed on Bruker MicroTof mass spectrometer.

LC–MS analyses were performed with an Agilent HPLC 1100 (Phenomenex Gemini Column 5μ C18 110 A 50×3.0 mm, eluted with 0→3′ 20% MeOH/H_2_O; 3→6′ 95% MeOH/H_2_O; 6→8′ 95% MeOH/H_2_O; 8.1→10′ 20%) and diode array detector in series with a Bruker MicroTof mass spectrometer.

Thin layer chromatography (TLC) was carried out on Merck silica gel 60 F254 plates using UV light and/or PMA, or KMnO_4_ for visualization. TLC data are given as the *R*_f_ value with the corresponding eluent system specified in brackets. Column chromatography was performed using Fluka silica gel 60. All reactions were carried out under dry and inert conditions (Argon atmosphere) unless otherwise stated.

***N*****-(4-Methoxybenzyloxy)phthalamide (10a)**. *N*-Hydroxyphthalimide (1.75 g, 10.7 mmol) and 4-methoxybenzyl alcohol (2.67 mL, 10.7 mmol) were stirred in 70 mL of CH_2_Cl_2_ at 0°C. Triphenyl phosphine (4.22 g, 16.1 mmol) was added followed by diisoproyl azodicarboxylate (3.2 mL, 16.1 mmol). The solution was stirred at room temperature for 24 h. The reaction was concentrated and recrystallized from 150 mL of boiling ethanol to give 1.88 g (61%) of **10a** as white crystals. *R*_f_ 0.67 (50% hexane/EtOAc). ^1^H NMR (500 MHz, CDCl_3_): *δ*=3.78 (3H, s, OMe), 5.13 (2H, s, CH_2_), 6.87 (2H, d, *J*=8.5 Hz, Ar-H), 7.30 (2H, d, *J*=8.1 Hz, Ar-H), 7.43 (2H, d, *J*=8.5 Hz, Ar-H), 7.69–7.71 (2H, m, Ar-H), 7.78–7.79 ppm (2H, m, H-Ar).

***N*****-(2,4-Dimethoxybenzyloxy)phthalamide (10b)**. *N*-Hydroxyphthalimide (1.75 g, 10.7 mmol) and 2,4-dimethoxybenzyl alcohol (1.75 g, 10.7 mmol) were stirred in 70 mL of CH_2_Cl_2_ at 0°C. Triphenyl phosphine (4.21 g, 16.1 mmol) was added followed by diisoproyl azodicarboxylate (3.2 mL, 16.1 mmol). The solution was stirred at room temperature for 24 h. The reaction was concentrated and recrystallized from 150 mL of boiling ethanol to give 1.94 g (58%) of **10b** as white crystals. *R*_f_ 0.50 (50% hexane/EtOAc). ^1^H NMR (500 MHz, CDCl_3_): *δ*=3.72 (3H, s, OCH_3_), 3.79 (3H, s, OCH_3_), 5.22 (2H, s, CH_2_), 6.40–6.45 (2H, m, Ar-H), 7.30 (1H, d, *J*=8.1 Hz, Ar-H), 7.69–7.71 (2H, m, Ar-H), 7.78–7.79 ppm (2H, m, Ar-H).

***O*****-(4-methoxybenzyl)hydroxylamine (11a)**. *N*-(4-Methoxybenzyloxy)phthalamide **10a** (1.88 g, 6.64 mmol) was stirred as a suspension in 60 mL of refluxing ethanol. *N*-methylhydrazine (0.38 mL, 7.30 mmol) was added, and the mixture was stirred at reflux for 1 h. The solution was concentrated to remove the ethanol. Ether (60 mL) was added, and the reaction mixture was allowed to stand at room temperature for 30 min. The resulting solid was filtered. The organic solution was concentrated to give 1.05 g (∼quant.) of **11a** as an oil. ^1^H NMR (500 MHz, CDCl_3_): *δ*=3.76 (3H, s, OCH_3_), 4.58 (2H, s, CH_2_), 5.34 (2H, bs, NH_2_), 6.85 (2H, d, *J*=8.4 Hz, Ar-H), 7.25 ppm (2H,d AB system, *J*=8.4 Hz, Ar-H).

***O*****-(2,4-Dimethoxybenzyl)hydroxylamine (11b)**. *N*-(2,4-dimethoxybenzyloxy)phthalamide **10b** (1.83 g, 5,84 mmol) was stirred as a suspension in 60 mL of refluxing ethanol. *N*-methylhydrazine (0.34 mL, 6.4 mmol) was added, and the mixture was stirred at reflux for 1 h. The solution was concentrated to remove the ethanol. Ether (60 mL) was added, and the reaction mixture was allowed to stand at room temperature for 30 min. The resulting solid was filtered. The organic solution was concentrated to give 1.37 g (∼quant.) of **11b** slightly contaminated with some phtalamide. *R*_f_ 0.69 (10% MeOH/CHCl_3_). ^1^H NMR (500 MHz, CDCI_3_): *δ*=3.77 (3H, s, OCH_3_), 3.78 (3H, s, OCH_3_), 4.64 (2H, s, CH_2_), 5.41 (2H, bs, NH_2_), 6.43–6.44 (2H, m, Ar-H), 7.19–7.22 ppm (1H, m, Ar-H).

**(4*R*,5*R*)-*N*-(2,4-dimethoxybenzyloxy)-5-(hydroxymethyl)-2,2-dimethyl-1,3-dioxolane-4-carboxamide (4b)**. Trimethylaluminum (2.0m solution in hexanes; 3.5 mL, 7 mmol) was added dropwise over 15 min to a stirred solution of **11b** (1.06 g, 5.84 mmol) and isopropilidene-erythronolactone (923 mg, 5.84 mmol) in CH_2_Cl_2_ (50 mL) cooled at −78°C. The clear yellowish solution was warmed to room temperature and stirred overnight. The clear solution was added cautiously in small portions to a vigorously stirred saturated aqueous NaHCO_3_ (100 mL, gas evolution occurs) and the mixture was stirred for 15 min. The aluminium salts formed were removed by filtration and washed with methanol and then with CH_2_Cl_2_. The organic layer of the biphasic filtrate was separated, the aqueous phase extracted with CH_2_Cl_2_ (3×20 mL) and the combined organic extracts were concentrated in vacuo. The residue was dissolved in a minimum amount of a warm mixture of hexane 20% in EtOAc and recrystallized upon cooling to 4°C. White crystals, 783 mg, 40%. *R*_f_ 0.13 (50% EtOAc/hexane). ^1^H NMR (500 MHz, CDCl_3_): *δ*=1.34 (3H, s, C(C*H*_3_)_2_), 1.41 (3H, s, C(C*H*_3_)_2_), 3.26–3.29 (1H, m, OH), 3.65–3.69 (1H, m, C*H*HOH), 3.77–3.79 (1H, m, CH*H*OH), 3.81 (3H, s, OCH_3_), 3.83 (3H, s, OCH_3_), 4.51–4.55 (1H, m, C*H*CH_2_OH), 4.66 (1H, d, *J*=7.5 Hz, COC*H*), 4.94 (2H, m, ArC*H*_2_), 6.47–6.49 (2H, m, Ar-H), 7.25 (1H, d, *J*=7.3 Hz, Ar-H), 8.91 ppm (1H, s, NH). ^13^C NMR (125 MHz, CDCl_3_): *δ*=24.3 (C(*C*H_3_)_2_), 26.6 (C(*C*H_3_)_2_), 55.46 (OCH_3_), 55.58 (OCH_3_), 61.4 (CH*C*H_2_OH), 73.6 (Ar*C*H_2_), 76.5 (*C*HCH_2_OH), 77.6 (CO*C*HCH), 98.6 (CH-Ar), 104.2 (CH-Ar), 110.2 (*C*(CH_3_)_2_), 115.5 (C-Ar), 132.9 (CH-Ar), 159.5 (CH_3_O*C*-Ar), 161.9 (CH_3_O*C*-Ar), 167.1 ppm (NH*C*OCH); LRMS (ES+): *m*/*z* (%) 342.5 (30) [*M*+H]^+^, 364.3 (100) [*M*+Na]^+^.

**2,2-Dimethyl-thiopropionic acid S-(2-hydroxy-ethyl) ester (13)**. Pivaloyl chloride (3.2 mL, 26 mmol) was added to a stirred solution of 2-mercaptoethanol (1.8 mL, 26 mmol) and triethylamine (3.6 mL, 26 mmol) in CH_2_Cl_2_, cooled at −78°C. The mixture was stirred at −78°C for 1 h, then allowed to warm to room temperature and stirred further for 1 h. Water (30 mL) was added, the organic layer was separated, and the aqueous phase extracted with CH_2_Cl_2_ (3×20 mL). The combined organic extracts were dried over Na_2_SO_4_ and concentrated in vacuo. The oily residue was purified by flash column chromatography (SiO_2_, hexane/EtOAc 90%→75%) to afford the title compound as colourless oil, 4.02 g, 95%. *R*_f_ 0.44 in (70% EtOAc/hexane). ^1^H NMR (500 MHz, CDCl_3_): *δ*=1.23 (9H, s, C(CH_3_)_3_), 2.62 (1H, bs, OH), 3.04 (2H, t, *J*=6.1 Hz SCH_2_), 3.72 ppm (2H, t, *J*=6.1 Hz, OCH_2_). ^13^C NMR (125 MHz, CDCl_3_): *δ*=27.8 (CH_3_), 31.9 (SCH_2_), 47.0 (CMe_3_), 63.2 (OCH_2_), 207.7 ppm (S*C*O).

**2,2-Dimethyl-thiopropionic acid** ***S*****-[2-(chloro-phenoxy-phosphoryloxy)-ethyl] ester (5a)**. Dichloro phenyl phosphonate (1.06 g, 5 mmol) was added dropwise into a cooled solution (−78°C) of **13** (0.81 g, 5 mmol) and TEA (0.70 mL, 5 mmol) in THF (20 mL). The reaction was left to warm to room temperature and stirred overnight. The white precipitate was filtered off and the solution was concentrated under reduced pressure, the crude residue (yellowish oil) was used for the next step without further purification. *R*_f_ 0.66 in (50% EtOAc/hexane). ^1^H NMR (500 MHz, CDCl_3_): *δ*=1.25 (9H, s, C(CH_3_)_3_), 3.24 (2H, t, *J*=6.4 Hz, CH_2_C*H*_2_S), 4.34–4.41 (2H, m, CH_2_C*H*_2_OP), 7.25–7.28 (3H, m, Ph-H), 7.39 ppm (2H, t, *J*=7.9 Hz, Ph-H). ^13^C NMR (125 MHz, CDCl_3_): *δ*=27.3 (C(*C*H_3_)_3_), 28.2, 28.1 (*C*H_2_S), 46.5 (*C*(CH_3_)_3_), 68.2 (d, *J*=7.20 Hz, *C*H_2_O), 120.3 (d, *J*=5.3 Hz, *orto-*CH-Ar), 126.3 (*meta-*CH-Ar), 130.0 (*para*-CH-Ar), 149.7 (d, *J*=8.9 Hz, C-Ar), 205.6 ppm (S*C*O). ^31^P NMR (202 MHz, CDCl_3_): *δ*=−0.66 ppm.

**2,2-Dimethyl-thiopropionic acid S-(2-{[5-(4-methoxy-benzyloxycarbamoyl)-2,2-dimethyl-[1,3]dioxolan-4-ylmethoxy]-phenoxy-phosphoryloxy}-ethyl) ester (26a)**. Compound **4a** (100 mg, 0.32 mmol) was dissolved in dry DCM (4.5 mL) and was added into a solution of **13** (151 mg, 0.45 mmol) in dry DCM (2.5 mL). The mixture was cooled to −78°C and followed by the addition of *N*-methylimidazole (0.076 mL, 0.96 mmol) with a syringe. The reaction was kept 15 min at −78°C and the 3 h at room temperature. The reaction was quenched adding methanol (2 mL). The residue was extracted with DCM and washed with HCl 0.1m (3×10 mL). The organic layer was dried over MgSO_4_, filtered, evaporated to dryness, and purified twice by flash chromatography. Eluting the first column with hexane/EtOAc 95%→60%. Eluting the second column with Chloroform 100%→Chloroform/MeOH 99.5%. The desired compound was obtained as colourless oil, 134 mg, 68%. ^1^H NMR (500 MHz, CDCl_3_): *δ*=1.14, 1.15 (9H, 2 s, C(CH_3_)_3_), 1.25, 1.26 (3H, 2 s, C(CH_3_)_2_), 1.34, 1,36 (3H, 2 s, C(CH_3_)_2_), 3.06 (2H, d, *J*=6.7 Hz, CH_2_S), 3.73 (3H, s, OCH_3_), 4.06–4.16 (3H, m, C*H*HOP+OC*H*_2_CH_2_S), 4.42–4.56 (3H, m, C*H*CH+CHC*H*+CHCH*H*OP), 4.74–4.83 (2H, m, ArCH_2_), 6.82 (2H, dd, *J*_1_=1.6 Hz, *J*_2_=8.7 Hz, Ar-H), 7.09 (1H, t, *J*=7.3 Hz, Ph-H), 7.15–7.17 (2H, m, Ar-H), 7.23–7.29 (4H, m, Ph-H), 8.70, 8.75 ppm (1H, 2 s, NH). ^13^C NMR (125 MHz, CDCl_3_): *δ*=24.5, 25.5 (C*C*H_3_), 26.5, 26.6 (C*C*H_3_), 27.0, 27.3 (C(*C*H_3_)_3_), 28.4, 28.5 (CH_2_S), 55.3 (OCH_3_), 66.66, 66.69, 66.71, 66.76 (CH*C*H_2_OP + PO*C*H_2_CH_2_S), 74.9, 75.0 (*C*HCH_2_OP), 75.8, 75.9 (CO*C*HCH), 78.2 (Ar*C*H_2_O), 110.7 (*C*(CH_3_)_2_), 113.9, 114.0 (Ph-CH), 120.1, 120.2 (Ph-CH), 125.2 (Ar-C), 126.78, 126.86 (Ph-C), 129.70 (Ar-CH), 131.1 (Ar-CH), 150.44, 150.50 (Ph-C), 160.1 (Ar-C), 165.5 (HN*C*O), 205.7 ppm (S*C*O). ^31^P NMR (202 MHz, CDCl_3_): *δ*=−6.94, −7.10 ppm; LRMS (ES+): *m*/*z* (%) 634.5 (100) [*M*+Na]^+^, 612.5 (50) [*M*+H]^+^.

**2,2-Dimethyl-thiopropionic acid S-(2-{[5-(2,4-dimethoxy-benzyloxycarbamoyl)-2,2-dimethyl-[1,3]dioxolan-4-ylmethoxy]-phenoxy-phosphoryloxy}-ethyl) ester (26b)**. NMI (0.07 mL, 0.87 mmol) was added into a cooled solution (−78°C) of **4b** (100 mg, 0.29 mmol) and **13** (140 mg, 0.41 mmol) in anhydrous DCM (7 mL). The reaction was stirred for 30 min at −78°C, then 1 h at room temperature. The solvent was removed under reduced pressure; the crude residue was dissolved in DCM (10 mL), washed with HCl 0.1 N (3×10 mL), dried over Na_2_SO_4_, and concentrated in vacuo. The crude oil was purified by chromatography (SiO_2_) eluting with EtOAc (35%→50%) in hexane. The title compound was isolated as colourless oil, 147 mg, 79%. ^1^H NMR (500 MHz, CDCl_3_): *δ*=1.14, 1.145 (9H, 2 s, C(CH_3_)_3_), 1.26, 1.27 (3H, 2 s, C(CH_3_)_2_), 1.37, 1.39 (3H, 2 s, C(CH_3_)_2_), 3.05 (2H, t, *J*=6.7 Hz, C*H*_2_S), 3.73 (3H, s, OCH_3_), 3.75 (3H, s, OCH_3_), 4.10–4.15 (3H, m, OC*H*_2_CH_2_S + CHCH*H*OP), 4.43–4.57 (3H, m, COC*H* + CHC*H*CH_2_OP + CHC*H*HOP), 4.80–4.87 (2, m, ArCH_2_), 6.39–6.43 (2H, m, Ph-H), 7.13–7.26 (5H, m, Ph-H + Ar-H), 8.80, 8.83 ppm (1H, 2 s, N*H*). ^13^C NMR (125 MHz, CDCl_3_): *δ*=24.5 (C(*C*H_3_)_2_), 26.68, 26.71 (C(*C*H_3_)_2_), 27.3 (C(*C*H_3_)_3_), 28.43, 28.48 (*C*H_2_S), 55.4, 55.5 (OCH_3_), 66.6, 66.7, 66.8, 66.9 (CH*C*H_2_OP + PO*C*H_2_CH_2_S), 73.5, 73.6 (Ar*C*H_2_), 74.95, 74.98 (*C*HCH_2_OP), 75.89, 75.94 (CO*C*HCH), 98.6 (Ar-CH), 104.1 (Ar-CH), 110.4 (C(CH_3_)_2_), 120.1, 120.2 (Ph-CH), 125.1 (Ph-CH), 129.7 (Ph-CH), 132.9 ppm (Ar-CH), ^31^P NMR (202 MHz, CDCl_3_): *δ*=−6.93, −7.09 ppm; LRMS (ES+): *m*/*z* (%) 664.5 (100) [*M*+Na]^+^, 642.5 (40) [*M*+H]^+^.

**2,2-Dimethyl-thiopropionic acid S-(2-{[5-(2,4-dimethoxy-benzyloxycarbamoyl)-2,2-dimethyl-[1,3]dioxolan-4-ylmethoxy]-phenoxy-phosphoryloxy}-ethyl) ester (3d). 26b** (115 mg, 0.18 mmol) was dissolved in a solution of 5% TFA in DCM (2 mL) and stirred at room temperature. After 15 min the colourless solution became deep purple and the starting material had completely reacted. The solution was concentrated under reduced pressure. The residue was taken in ether (5 mL), the white precipitate was filtered off, and the filtrate was concentrated in vacuo. The residue was redissolved in MeOH (5 mL), and the second precipitate formed was filtered. The filtrate was purified by chromatography eluting the silica with MeOH 0%→10% in DCM. The mix SATE prodrug **3d** was obtained as colourless oil, 56 mg (63%). ^1^H NMR (500 MHz, CDCl_3_): *δ*=1.15 (9H, s, C(C*H*_3_)_3_), 1.28 (3H, s, C(C*H*_3_)_2_),1.44, 1.45 (3H, 2 s, C(C*H*_3_)_2_), 3.07 (2H, dd, *J*=12.4, 6.4 Hz, CH_2_S), 4.09–4.22 (3H, m, C*H*_2_CH_2_S + CHC*H*HOP), 4.29–4.34 (1H, m, CH*H*OP), 4.52 (1H, bs, C*H*CH_2_OP), 4.66 (1H, d, *J*=7.6 Hz, COC*H*CH), 7.09–7.16 (3H, m, Ph-H), 7.27 (2H, t, *J*=7.9 Hz, Ph-H), 9.00, 9.09 ppm (1H, 2 s, N*H*). ^13^C NMR (125 MHz, CDCl_3_): *δ*=24.55, 24.57 (C(*C*H_3_)_2_), 26.63, 26.68 (C(*C*H_3_)_2_), 27.3 (C(*C*H_3_)_3_), 28.33, 28.41 (*C*H_2_S), 66.53, 66.58, 66.69, 66.74, 66.87, 66.92, 67.13, 67.17 (*C*H_2_OP + *C*H_2_CH_2_S), 74.68, 74.79 (*C*HCH_2_OP), 75.75, 75.80 (CO*C*HCH), 111.5 (*C*(CH_3_)_2_), 120.10, 120.17 (Ph-CH), 125.36, 125.41 (Ph-CH), 129.79 (Ph-CH), 150.4 (Ph-C), 165.2 (HN*C*OCH)), 205.9 ppm (S*C*O); ^31^P NMR (202 MHz, CDCl_3_): *δ*=−7.03, −7.06 ppm. LRMS (ES+): *m*/*z* 514.2 (100) [*M*+Na]^+^; HRMS (ES+) required for C_20_H_31_N_1_O_9_PS 492.1452, found 492.1429. LC-M: R_t_ 8.3 min; *m*/*z* 492; purity 80% by UV and TIC traces.

**Diisopropylamino dichloro phosphine (22)**. A solution of diisopropylamine (10.5 mL, 75 mmol) in THF (30 mL) was added dropwise into a vigorously stirred solution of PCl_3_ (3.25 mL, 32.5 mmol) in THF (30 mL) at −78°C, under atmosphere of Argon. The white suspension was stirred at room temperature for 2 h. The hydrochloride salt was filtered off and washed with THF (15 mL). The filtrate was concentrated to a colourless oil, with the rotary evaporator, and was then purified by distillation under vacuum (76–78°C, 5 mbar ca) avoiding any contact with air. The title compound was obtained as colourless liquid (3.46 g, 53%) which solidifies at 4°C. ^1^H NMR (500 MHz, CDCl_3_): *δ*=1.20, 1.21 (12H, 2 s, 2×(C*H*_3_)_2_CH), 3.86 ppm (2H, bs, 2×C*H*(CH_3_)_2_); ^31^P NMR (202 MHz, CDCl_3_): *δ*=169.6 ppm.

**2,2-Dimethyl-thiopropionic acid S-(2-{diisopropylamino-[2-(2,2-dimethyl-propionylsulfanyl)-ethoxy]-phosphanyloxy}-ethyl) ester (6a)**. A solution of S-pivaloyl thioethanol **13** (1.62 g, 10 mmol) and triethylamine (3.06 mL, 22 mmol) in THF (35 mL) was added dropwise over 1.5 h at −78°C into a solution of diisopropylamino dichloro phosphine **22** (1.01 g, 9 mmol) in THF (35 mL). The white suspension was stirred for 2 h at room temperature, and then it was filtered to remove the triethylamine hydrochloride salt. The filtrate was concentrated under reduced pressure affording a white syrup. The residue was further purified by treating it with hexane and filtering the white precipitate formed. The title compound was used as crude for the next step. ^1^H NMR (500 MHz, CDCl_3_): *δ*=1.09, 1.10 (12H, 2 s, 2×CH(C*H*_3_)_2_), 1.16, 1.17 (18H, 2 s, 2×C(C*H*_3_)_3_), 3.00–3.06 (4H, m, 2×C*H*_2_S), 3.46–3.71 ppm (6H, m, 2×(C*H*_2_O) + 2×(C*H*(CH_3_)_2_); ^31^P NMR (202 MHz, CDCl_3_): *δ*=147.2 ppm.

**2,2-dimethyl-thiopropionic acid S-(2-{[5-(2,4-dimethoxy-benzyloxycarbamoyl)-2,2-dimethyl-[1,3]dioxolan-4-ylmethoxy]-[2-(2,2-dimethyl-propionylsulfanyl)-ethoxy]-phosphoryloxy}-ethyl) ester (27)**. Tetrazole (3.3 mL of solution 0.45m in CH_3_CN) was added to a suspension of **6a** (340 mg, 0.75 mmol) and **4b** (171 mg, 0.50 mmol) in THF. After the addition the mixture became a clear solution and was stirred for 1.5 h at room temperature. The flask was cooled to −78°C with a dry-ice/acetone bath and followed by the addition of a solution of *tert*-butyl hydroperoxide in water (0.15 mL, 1.20 mmol). The reaction was kept at −78°C for 15 min, then warmed to room temperature and stirred for a further 30 min. A solution of Na_2_SO_3_ 10% in water (1.2 mL) was added to destroy the excess of peroxide. The solution was diluted with DCM (20 mL), washed with water (10 mL), and dried over Na_2_SO_4_. The organic solution was concentrated under reduced pressure to afford a colourless oil which was purified by chromatography. SiO_2_ eluted with hexane 60%→30% in EtOAc. Compound **27** was obtained as viscous oil, 229 mg, 64%. ^1^H NMR (500 MHz, CDCl_3_): *δ*=1.16 (18H, s, 2×C(CH_3_)_3_), 1.28 (3H, s, C(CH_3_)_2_), 1.41 (3H, s, C(CH_3_)_2_), 3.07 (4H, t, *J*=6.6 Hz, 2×CH_2_S), 3.75 (3H, s, OCH_3_), 3.77 (3H, s, OCH_3_), 3.96–4.07 (5H, m, 2×POC*H*_2_CH_2_S+ CHCH*H*OP), 4.34–4.37 (1H, m, C*H*HOP), 4.55 (2H, bs, C*H*C*H*CH_2_OP), 4.85 (2H, AB syst., *J*=11.0 Hz, ArCH_2_), 6.40–6.43 (2H, m, Ar-H), 7.20 (1H, s, Ar-H), 8.83 ppm (1H, bs, NH). ^13^C NMR (125 MHz, CDCl_3_): *δ*=24.6 (C(*C*H_3_)_2_), 26.7 (C(*C*H_3_)_2_), 27.3 (C(*C*H_3_)_3_), 28.5, 28.55 (*C*H_2_S), 46.5 (*C*(CH_3_)_3_), 55.4, 55.6 (OCH_3_), 66.1, 66.2, 66.3 (PO*C*H_2_ + CH*C*H_2_OP), 73.5 (ArCH_2_), 75.0 (*C*HCH_2_OP), 75.96, 76.02 (CO*C*H), 98.6 (Ar-CH). 104.2 (Ar-CH), 110.7 (*C*(CH_3_)_2_), 115.7 (Ar-CH), 132.9 (Ar-C), 159.4 (Ar-C), 161.8 (Ar-C), 165.2 (HN*C*O), 205.7 ppm (S*C*O). ^31^P NMR (202 MHz, CDCl_3_): *δ*=−2.05 ppm; LRMS (ES+): *m*/*z* (%) 710 (15) [*M*+H]^+^, 727 (100) [*M*+H_2_O]^+^, 732 (90) [*M*+Na]^+^.

**2,2-Dimethyl-thiopropionic acid S-{2-[[2-(2,2-dimethyl-propionylsulfanyl)-ethoxy]-(5-hydroxycarbamoyl-2,2-dimethyl-[1,3]dioxolan-4-ylmethoxy)-phosphoryloxy]-ethyl} ester (3a)**. TFA (0.040 mL) was added into a solution of **27** (157 mg, 0.22 mmol) in DCM (4 mL). The reaction was stirred at room temperature until the complete disappearance of the starting material was observed by TLC (5% MeOH in DCM). The mixture was diluted with MeOH (5 mL) and the white precipitate was filtered off. The filtrate was concentrated in vacuo and purified by chromatography. SiO_2_ eluted with MeOH 0%→4% in DCM. The *bis*SATE phosphate diester prodrug **3a** was obtained as colourless oil, 91 mg, 73%. ^1^H NMR (500 MHz, CDCl_3_): *δ*=1.17, 1.17 (18H, 2 s, C(C*H*_3_)_3_), 1.30 (3H, s, C(C*H*_3_)_2_), 1.47 (3H, s, C(C*H*_3_)_2_), 3.06 (2H, t, *J*=6.8 Hz, CH_2_S), 3.09 (2H, t, *J*=6.7 Hz, CH_2_S), 4.00–4.05 (2H, m, CHC*H*_2_OP), 4.06–4.20 (4H, m, 2×POC*H*_2_CH_S_), 4.49–4.53 (1H, m, C*H*CH_2_OP), 4.68 (1H, d, *J*=7.5 Hz, COC*H*), 7.72 (1H, bs, O*H*), 8.90 ppm (1H, s, N*H*). ^13^C NMR (125 MHz, CDCl_3_): *δ*=24.6 (C(*C*H_3_)_2_), 26.7 (C(*C*H_3_)_2_), 27.32, 27.33 (C(*C*H_3_)_3_), 28.4 (CH_2_S), 46.6 (*C*(CH_3_)_3_), 65.6, 66.36, 66.40, 66.75, 66.79 (CH*C*H_2_OP + PO*C*H_2_), 74.96 (*C*HCH_2_OP), 75.85 (CO*C*H), 110.7 (*C*(CH_3_)_2_), 165.25 ppm (HN*C*O). ^31^P NMR (202 MHz, CDCl_3_): *δ*=−2.00 ppm; LRMS (ES+): *m*/*z* (%) 560 (100) [*M*+H]^+^, 561 (25) [*M*+H]^+^, 1119 (35) [2*M*+H]^+^. HRMS (ES+) required for C_21_H_39_NO_10_PS_2_: 560.1748, found: 560.1747. LC-M: *R*_t_ 8.6 min; *m*/*z* 560; purity 99% by TIC trace.

**Phenyl methoxyalaninyl phosphochloridate (5c)**. The phenyl dichlorophosphate (0.400 mL, 2.69 mmol) and l-alanine methyl ester hydrochloride (375 mg, 2.69 mmol) were suspended in DCM (10 mL). Anhydrous triethylamine (0.750 mL, 5.38 mmol) was added dropwise at −78°C and after 15 min the reaction mixture was left to warm to room temperature. The formation of the phosphochloridate was monitored by ^31^P NMR. After 14 h the solvent was removed under reduced pressure and the resulting slurry was purified by flash chromatography on silica gel (70% EtOAc in hexane). Alternatively the triethylamine salt was precipitated with ether and filtered off, and the crude was used without further purification in the next step. The entitled product was identified as slightly yellow oil, 320 mg, 43%. ^1^H NMR (500 MHz, CDCl_3_): *δ*=1.08–1.11 (3H, m, CHC*H*_3_), 3.36, 3.38 (3H, 2 s, OC*H*_3_), 3.69–3.82 (1H, bs, N*H*), 3.88–3.97 (1H, m, C*H*CH_3_), 6.81–6.85 (3H, m, Ph-H), 6.94–6.97 ppm (2H, m, Ph-H). ^31^P NMR (300 MHz, CDCl_3_): *δ*=7.5, 7.2 ppm. LRMS (ES+): *m*/*z* (%) 300 (100) [*M*+Na]^+^.

**4*-*Nitro-phenyl methoxyalaninyl phosphochloridate (5d)**. Same procedure as for compound **5c**, starting from *p*-nitro phenyl dichlorophosphate (367 mg, 1.43 mmol), l-alanine hydrochloride methyl ester (200 mg, 1.43 mmol), and triethylamine (0.400 mL, 2.86 mmol) in DCM (10 mL). The title compound was used as crude after precipitation with ether and filtration of the triethylamine salt. ^31^P NMR (202 MHz, CDCl_3_): *δ*=7.57, 7.30 ppm.

**2-{[5-(4-Methoxy-benzyloxycarbamoyl)-2,2-dimethyl-[1,3]dioxolan-4-ylmethoxy]-phenoxy-phosphorylamino}-propionic acid methyl ester (25a)**. To a cooled (−78°C) solution of **4a** (31 mg, 0.1 mmol) and **5c** (111 mg, 0.4 mmol) in dry THF (2 mL), NMI (0.048 mL, 0.6 mmol) was added dropwise with a syringe over 1 min. The reaction was stirred for 5 min at −78°C, then 6 h at room temperature and it was kept for the weekend in the freezer. TLC still revealed presence of the starting materials and the mixture was stirred for further 8 h at room temperature. The solvent was removed under reduced pressure. The crude yellowish residue was taken in DCM (10 mL) and washed with HCl 0.1m (2×10 mL). The organic phase was dried over Na_2_SO_4_, filtered, and concentrated under reduced pressure. The resulting colourless oil was purified by chromatography (SiO_2_, MeOH 0%→2% in DCM). Compound **25a** was obtained as a yellow oil, 44 mg, 79%. ^1^H NMR (500 MHz, CDCl_3_): *δ*=1.28–1.41 (9H, m, CHC*H*_3_ + C(C*H*_3_)_2_), 3.69, 3.70 (3H, 2 s, CO_2_C*H*_3_), 3.81, 3.83 (3H, 2 s, PhOC*H*_3_), 4.05–4.20 (2H, m, C*H*CH_3_ + CHCH*H*OP), 4.41–4.50 (1H, m, CHC*H*HOP), 4.57–4.69 (2H, m, COC*H* + C*H*CH_2_OP), 4.88 (2H, s, ArCH_2_), 6.90 (2H, t, *J*=8.4 Hz, Ph-H), 7.12–7.16 (1H, m, Ph-H), 7.20–7.22 (2H, m, Ph-H), 7.27–7.38 (4H, m, Ar-H), 8.83, 9.13 ppm (1H, 2 s, N*H*). ^31^P NMR (202 MHz, CDCl_3_): *δ*=2.08, 2.58 ppm.

**2-{[5-(2,4-Dimethoxy-benzyloxycarbamoyl)-2,2-dimethyl-[1,3]dioxolan-4-ylmethoxy]-phenoxy-phosphorylamino}-propionic acid methyl ester (25b)**. Same procedure as **25a**. Starting from **4b** (150 mg, 0.44 mmol), **5c** (3.51 mL of solution 0.5m in DCM, 1.76 mmol) and NMI (0.210 mL, 2.64 mmol). Purified by chromatography eluting the silica with MeOH 0→2% in DCM. The title compound was obtained as colourless oil, slightly contaminated with starting material, 84 mg, 33%. ^1^H NMR (500 MHz, CDCl_3_): *δ*=1.20–1.33 (9H, m, CHC*H*_3_ + C(C*H*_3_)_2_), 3.59, 3.60 (3H, 2 s, CO_2_CH_3_), 3.72, 3.74 (6H, 2 s, OCH_3_), 3.94–4.08 (2H, m, CH*H*OP, C*H*CH_3_) 4.33–4.43 (1H, m, C*H*HOP), 4.49–4.59 (2H, m, COC*H*C*H*CH_2_), 4.82–4.89 (2H, m, ArCH_2_), 6.38–6.40 (2H, m, Ph-H), 7.02–7.24 (4H, m, Ar-H + Ph-H), 8.84, 9.06 ppm (1H, 2 s, N*H*). ^13^C NMR (125 MHz, CDCl_3_): *δ*=20.9, 21.0, 21.1 (C(*C*H_3_)_3_), 24.5, 24.6 (C(*C*H_3_)_2_), 26.5, 26.7 (C(*C*H_3_)_2_), 49.9, 50.1 (*C*HCH_3_), 52.3, 52.4 (CO_2_*C*H_3_), 55.4, 55.5 (PhO*C*H_3_), 64.9, 65.0, 65.2, 65.24 (CH_2_OP), 73.5 (ArCH_2_), 74.9, 75.3 (CH*C*HCH_2_) 76.0, 76.11, (CO*C*HCH), 98.5 (Ar-CH), 104.1 (Ar-CH), 120.27, 120.32, 120.36, 120.4 (Ph-CH), 124.5, 124.7 (Ph-CH), 129.5, 129.6 (Ph-CH), 132.8 ppm (Ar-CH). In the ^13^C NMR all quaternary carbons are absent (7 in total). ^31^P NMR (202 MHz, CDCl_3_): *δ*=2.58, 2.78 ppm.

**2-[(5-Hydroxycarbamoyl-2,2-dimethyl-[1,3]dioxolan-4-ylmethoxy)-phenoxy-phosphorylamino]-propionic acid methyl ester (3b). 25b** (84 mg, 0.14 mmol) was dissolved in a solution of TFA 2% in DCM (2 mL). The reaction was stirred 15 min at room temperature; until the complete disappearance of the starting material was observed by TLC (2% MeOH in DCM) and the solution became deep purple. The mixture was diluted with DCM (5 mL), treated with diethyl ether (5 mL). The white precipitate was filtered and the solution was concentrated under reduced pressure and purified by chromatography. SiO_2_ eluted with MeOH 0%→7.5% in DCM. The phosphoramidate **3b** was obtained as colourless oil, 19 mg, 22%. ^1^H NMR (500 MHz, CDCl_3_): *δ*=1.26–1.30 (6H, m, C(C*H*_3_)_2_), 1.42, 1.44 (3H, 2 s, CHC*H*_3_), 3.64, 3.65 (3H, 2 s, OC*H*_3_), 3.92–4.22 (4H, m, C*H*CH_3_ + C*H*_2_OP + O*H*), 4.49–4.54 (1H, m, C*H*CH_2_OP), 4.68 (1H, t, *J*=7.4 Hz, COC*H*CH), 7.07–7.10 (1H, m, Ph-H), 7.13–7.16 (2H, m, Ph-H), 7.23–7.26 (2H, m, Ph-H), 8.32 (1H, bs, N*H*), 8.96 ppm (1H, bs, N*H*). ^13^C NMR (125 MHz, CDCl_3_): *δ*=20.87, 20.93 (CH*C*H_3_), 24.57, 24.59 (C(*C*H_3_)_2_), 26.39, 26.68 (C(*C*H_3_)_2_), 50.0, 50.2 (*C*HCH_3_), 52.6 (O*C*H_3_), 64.9, 65.4 (*C*H_2_OP), 74.8, 75.4 (*C*HCH_2_OP), 75.79, 75.87, 75.96, 76.05 (CO*C*HCH), 109.7 (*C*(CH_3_)_2_), 120.2, 120.3 (Ph-CH), 124.9, 125.0 (Ph-CH), 129.7 (Ph-CH), 149.5 (Ph-C), 165.1 ppm (HN*C*OCH), 173.8 (*C*O_2_CH_3_); ^31^P NMR (202 MHz, CDCl_3_): *δ*=2.49, 2.65 ppm; LRMS (ES+) *m*/*z* (%) 433 (100) [*M*+H]^+^, 434 (23) [*M*+H]^+^, 455 (95) [*M*+Na]^+^, 456 (19) [*M*+Na]^+^. HRMS (ES+) required for C_17_H_26_N_2_O_9_P 433.1370, found 433.1397. LC-M: *R*_t_ 7.6 min; *m*/*z* 433; purity 99% by UV and TIC traces.

**2-[[5-(2,4-Dimethoxy-benzyloxycarbamoyl)-2,2-dimethyl-[1,3]dioxolan-4-ylmethoxy]-(4-nitro-phenoxy)-phosphorylamino]-propionic acid methyl ester (25c)**. NMI (0.210 mL, 2.64 mmol) was added dropwise into a solution of **4b** (150 mg, 0.44 mmol), **5d** (425 mg, 1.32 mmol) in dry DCM (10 mL) at −78°C under Argon. The reaction was at stirred room temperature for 48 h. The solution was washed with HCl 0.1n (3×10 mL). The organic phase was dried over Na_2_SO_4_, concentrated under reduced pressure, and purified by chromatography. SiO_2_ eluted with MeOH 0%→0.1% in Chloroform. The title compound was obtained as yellowish oil, slightly contaminated of starting alcohol **4b**, 60 mg (29%) and was used for the next step without further purification. ^1^H NMR (500 MHz, CDCl_3_): *δ*=1.30, 1.32 (6H, 2 s, C(CH_3_)_2_), 1.42, 1.46 (3H, 2 s, CHC*H*_3_), 3.68 (3H, s, OCH_3_), 4.04–4.09 (1H, m, C*H*CH_3_), 4.15–4.23 (2H, m, C*H*_2_OP), 4.53 (1H, bs, C*H*CH_2_OP), 4.70 (1H, d, *J*=7.5 Hz, COC*H*CH), 6.42 (2H, m, Ar-C*H*_2_), 7.31 (2H, m, Ar-H), 8.16 (2H, m, Ar-H), 8.93 ppm (1H, bs, N*H*). ^31^P NMR (202 MHz, CDCl_3_): *δ*=2.30, 1.77 ppm; LRMS (ES+): *m*/*z* (%) 626.5 (100) [*M*+H]^+^, 645.6 (85) [*M*+NH_4_]^+^, 650.5 (75) [*M*+Na]^+^.

**2-[(5-Hydroxycarbamoyl-2,2-dimethyl-[1,3]dioxolan-4-ylmethoxy)-(4-nitro-phenoxy)-phosphorylamino]-propionic acid methyl ester (3c)**. The protected hydroxamate **25c** (14 mg, 0.022 mmol) was stirred in a solution of 1% TFA in DCM (2 mL). After 15 min the solution turned deep purple and the TLC (5% MeOH in DCM) showed the complete disappearance of the starting material. The solution was concentrated under reduced pressure. The residue taken in MeOH (2 mL) the white precipitate was filtered and the solution was purified by chromatography eluting the silica with MeOH 0%→5% in DCM. The *p*-nitro phosphoramidate **3c** was obtained as yellowish gum, 5 mg, 11%. ^1^H NMR (500 MHz, CDCl_3_): *δ*=1.30, 1.32 (6H, 2 s, C(CH_3_)_2_), 1.42, 1.46 (3H, 2 s, CHC*H*_3_), 3.68 (3H, s, OCH_3_), 4.04–4.09 (1H, m, C*H*CH_3_), 4.15–4.23 (2H, m, C*H*_2_OP), 4.53 (1H, bs, C*H*CH_2_OP), 4.70 (1H, d, *J*=7.5 Hz, COC*H*CH), 7.31 (2H, d, *J*=8.7 Hz, Ar-H), 8.16 (2H, d, *J*=9.0 Hz, Ar-H), 8.93 ppm (1H, bs, N*H*); ^13^C NMR (125 MHz, CDCl_3_): *δ*=20.0 (CH*C*H_3_), 24.25 (C(*C*H_3_)_2_), 26.67 (C(*C*H_3_)_2_), 50.0, 50.2 (*C*HCH_3_), 53.5 (O*C*H_3_), 65.1, 65.2 (*C*H_2_OP), 75.5, 75.6 (*C*HCH_2_OP), 77.6 (CO*C*HCH), 110.5 (*C*(CH_3_)_2_), 120.8, 120.9 (Ph-CH), 125.6, (Ph-CH), 130.7 (Ph-C-NO_2_), 142.6 (Ph-C), 166.0 (HN*C*OCH), 173.8 ppm (*C*O_2_CH_3_); ^31^P NMR (202 MHz, CDCl_3_): *δ*=2.10, 1.90 ppm; LRMS (ES+): *m*/*z* 499.5 ([*M*+Na]^+^ 100%), 477.5 ([*M*+H]^+^, 40%); LRMS (ES+) *m*/*z* (%) 478 (10) [*M*+H]^+^, 479 (24) [*M*+H]^+^, 500 (18) [*M*+Na]^+^, 537 (6) [*M*+K]^+^. HRMS (ES+) required for C_17_H_25_N_3_O_11_P 478.1221, found 478.1218. LC-M: *R*_t_ 7.6 min; *m*/*z* 478; purity 80% by UV and TIC traces.

**2-Chloro-4*H*-benzo[1,3,2]dioxaphosphinine (6b)**. A solution of triethylamine (11.8 mL, 84.6 mmol) in dry ether (70 mL) was added dropwise over 1.5 h into a solution of PCl_3_ (6.2 mL, 44.3 mmol) and 2-hydroxy benzyl alcohol (5 g, 40.3 mmol) in dry ether (100 mL) at −78°C. The white suspension was vigorously stirred at room temperature for 1.5 h. The hydrochloride salt was filtered off and the solution was first concentrated at room temperature with the rotary evaporator and then distilled under reduced pressure (5 mbar, 80–93°C), avoiding any contact with air. The cyclosal chloro phosphine was obtained as colourless liquid (2.56 g, 34%). ^1^H NMR (500 MHz, CDCl_3_): *δ*=5.07 (1H, dd, *J*_1_=14.2, *J*_2_=9.6 Hz, Ar-C*H*HO), 5.49 (1H, dd, *J*_1_=14.4, *J*_2_=1.9 Hz, Ar-CH*H*O), 7.01–7.03 (2H, m, Ar-H), 7.15 (1H, t, *J*=7.5 Hz, Ar-H), 7.31 ppm (1H, t, *J*=7.5 Hz, Ar-H). ^13^C NMR (125 MHz, CDCl_3_): *δ*=61.13, 61.14 (Ar*C*H_2_O), 119.47, 119.49 (Ar-CH), 121.44, 121.54 (Ar-C), 124.04 (Ar-CH), 125.84 (Ar-CH), 129.30 (Ar-CH), 146.13, 146.17 (Ar-C). ^31^P NMR (202 MHz, CDCl_3_): *δ*=139.95 ppm.

**2,2-Dimethyl-5-(2-oxo-2,3-dihydro-2,5-benzo[1,4,2]dioxaphosphinin-2-yloxymethyl)-[1,3]dioxolane-4-carboxylic acid (2,4-dimethoxy-benzyloxy)-amide (28). 6b** (166 mg, 0.88 mmol) was added over 5 min to a solution of **4b** (150 mg, 0.44 mmol) and DIPEA (0.152 mL, 0.88 mmol) in dry CH_3_CN (10 mL) cooled with an ice bath. The reaction was kept 1 h at 0°C, until the TLC (10% MeOH in DCM) showed complete disappearance of the starting alcohol; then a solution of *tert*-butylhydroperoxide 70% in H_2_O (0.118 mL) was added. The mixture was stirred for further 2 h at room temperature. Na_2_SO_3_ 10% in water (0.055 mL) was added to destroy the excess of peroxide. The reaction was diluted with DCM (10 mL) and washed with water/brine (10 mL). The organic phase was dried over Na_2_SO_4_, concentrated to dryness, and purified by chromatography. SiO_2_ eluted with DCM 10%→5% in EtOAc. The title compound was obtained as white foam, 84 mg, 37%. ^1^H NMR (500 MHz, CDCl_3_): *δ*=1.27 (3H, s, C(C*H*_3_)_2_), 1.38, 1.41 (3H, 2 s, C(C*H*_3_)_2_), 3.75 (6H, 2 s, OCH_3_), 4.04–4.16 (1H, m, CHC*H*HOP), 4.46–4.57 (3H, m, COC*H*C*H*CH*H*OP), 4.75 (1H, AB syst., *J*_1_=11.0, *J*_2_=28.4 Hz, ArC*H*HO), 4.80 (1H, AB syst., *J*_1_=11.0, *J*_2_=32.9 Hz, ArCH*H*O), 5.16–5.27 (1H, m, POC*H*HAr), 5.33–5.39 (1H, m, POCH*H*Ar), 6.38–6.42 (2H, m, Ar-H), 6.92–7.04 (3H, m, Ar-H), 7.13–7.22 (2H, m, Ar-H), 8.71, 8.78 ppm (1H, 2 s, N*H*). ^13^C NMR (125 MHz, CDCl_3_): *δ*=24.55, 24.58 (C(*C*H_3_)_2_), 26.66, 26.73 (C(*C*H_3_)_2_), 55.44, 55.55, 55.58 (OCH_3_), 66.60, 66.66, 66.70 (CH*C*H_2_OP), 68.54, 68.60, 68.72 (PO*C*H_2_Ar), 73.53, 73.57 (ArCH_2_O), 74.90, 75.02 (*C*HCH_2_OP), 75.81, 75.87 (CO*C*H), 98.55, 98.57 (Ar-CH), 104.16, 104.19 (Ar-CH), 110.75 (*C*(CH_3_)_2_), 115.63 (Ar-CH), 118.61, 118.68, 118.77, 118.84 (Ar-CH), 120.66 (Ar-C), 124.13, 124.18 (Ar-CH), 125.23, 125.33 (Ar-CH), 129.60, 129.70 (Ar-CH), 132.80, 132.88 (Ar-C), 150.20 (Ar-C), 159.39, 159.43 (Ar-C), 161.83, 161.86 (Ar-C) 165.07, 165.22 ppm (HN*C*O). ^31^P NMR (202 MHz, CDCl_3_): *δ*=−9.63, −9.79 ppm. LRMS (ES+): *m*/*z* (%) 532 (100) [*M*+Na]^+^, 533 (22) [*M*+Na]^+^. HRMS (ES+) required for C_23_H_28_NNaO_10_P 532.1343, found 532.1316.

***Cyclo*****(saligenyl)-(5-(hydroxycarbamoyl)-2,2-dimethyl-1,3-dioxolan-4-yl)methyl phosphate ester (3f)**. Compound **28** (79 mg, 0.155 mmol) was stirred in a solution of TFA 2% in DCM (2 mL). The reaction was monitored by TLC (5% MeOH/DCM). The solution was concentrated under reduced pressure, suspended in MeOH (2 mL), and filtrate. The collected filtrate was concentrated and purified by chromatography eluting the silica with MeOH 0→5% in DCM. The cycloSal prodrug **3f** was obtained as white foam, 39 mg, 69%. *R*_f_ 0.25 in (5% MeOH/DCM). ^1^H NMR (500 MHz, CDCl_3_): *δ*=1.38 (3H, s, C(C*H*_3_)_2_), 1.52, 1.55 (3H, 2 s, C(C*H*_3_)_2_), 4.41–4.46 (2H, m, CHC*H*_2_OP), 4.60–4.64 (1H, m, C*H*CH_2_OP), 4.76–4.78 (1H, m, COC*H*), 5.31–5.46 (2H, m, OCH_2_Ar), 7.09–7.11 (2H m, Ar-H), 7.17 (1H, t, *J*=7.5 Hz, Ar-H), 7.35 (1H, t, *J*=7.6 Hz, Ar-H), 8.93–9.02 ppm (1H, bs, N*H*). ^13^C NMR (125 MHz, CDCl_3_): *δ*=24.45, 24.58 (C(*C*H_3_)_2_), 26.47, 26.63 (C(*C*H_3_)_2_), 66.67, 66.72, 66.77 (CH*C*H_2_OP), 68.83, 68.88, 68.94 (PO*C*H_2_Ar), 74.45, 74.67 (*C*HCH_2_OP), 75.66, 75.72 (CO*C*HCH), 110.87, 110.95 (*C*(CH_3_)_2_), 118.66, 118.73, 118.75, 118.83 (Ar-CH), 120.57, 120.65, 120.72 (Ar-C), 124.40 (Ar-CH), 125.33, 125.41 (Ar-CH), 129.75, 129.78 (Ar-CH), 149.92, 149.94, 149.97, 150.0 (Ar-C), 165.7, 166.0 ppm (HN*C*O). ^31^P NMR (202 MHz, CDCl_3_): *δ*=−9.74, −9.83 ppm. LRMS (ES+): *m*/*z* (%) 360 (100) [*M*+H]^+^, 379 (85) [*M*+Na]^+^. HRMS (ES+): required 382.0662 for C_14_H_18_NNaO_8_P, found 382.0659. LC-M: R_t_ 7.2 min; *m*/*z* 360; purity 99% by UV and TIC traces.

***Tris*****((pivaloyloxy)methyl) phosphate (18)**. Chloromethyl pivalate (6.05 mL, 41.7 mmol) and NaI (4.84 g, 32.1 mmol) were added into a solution of trimethyl phosphate (1.24 mL, 10.7 mmol) in CH_3_CN (9 mL). The solution was refluxed (80°C) for 3 days, cooled to room temperature and diluted with diethyl ether (100 mL). The organic solution was washed with water (3×20 mL), dried over Na_2_SO_4_, concentrated and purified by chromatography eluting the column with 30% EtOAc in hexane. Compound **18** was obtained as colourless oil, 3.31 g (70%). *R*_f_ 0.47 (30% EtOAc/hexane). ^1^H NMR (500 MHz, CDCl_3_): *δ*=1.25 (27H, d, *J*=1.53 Hz, 3×C(C*H*_3_)_3_), 5.68 ppm (6H, dd, *J*=1.4, 13.8 Hz, 3×OC*H*_2_O). ^13^C NMR (125 MHz, CDCl_3_): *δ*=26.8 (C(*C*H_3_)_3_), 38.8 (*C*(CH_3_)_3_), 82.77 (d, *J*=5.2 Hz, O*C*H_2_O), 176.6 ppm (*C*OC(CH_3_)_3_). ^31^P NMR (202 MHz, CDCl_3_): *δ*=−5.1 ppm.

***Bis*****((pivaloyloxy)methyl) hydrogen phosphate (20)**. Tris((pivaloyloxy)methyl) phosphate **18** (3.31 g, 7.51 mmol) was dissolved in piperidine (20 mL) and stirred overnight at room temperature. The reaction was monitored by ^31^P NMR, and when the spectrum showed the complete disappearance of the starting material the solution was concentrated under reduced pressure until constant weight. The crude was dissolved in water (20 mL) treated with Dowex H^+^ 50 W8 (100/200 mesh) until the pH resulted acidic. The suspension was stirred at room temperature for 30 min. The resin was filtered and washed with H_2_O. The collected filtrate was concentrated and freeze-dried affording a white solid, 2.07 g (84%). ^1^H NMR (500 MHz, CDCl_3_): *δ*=1.25 (18H, s, 2×C(C*H*_3_)_3_), 5.66 (4H, d, *J*=13.9 Hz, 2×OC*H*_2_O), 10.4 ppm (1H, bs, OH). ^13^C NMR (125 MHz, CDCl_3_): *δ*=26.8 (C(*C*H_3_)_3_), 38.8 (*C*(CH_3_)_3_), 82.7 (d, *J*=5.29 Hz, O*C*H_2_O), 176.9 ppm (*C*OC(CH_3_)_3_). ^31^P NMR (202 MHz, CDCl_3_): *δ*=−2.7 ppm. LRMS (ES+): *m*/*z* (%) 327(45) [*M*+H]^+^, 344 (100) [*M*+Na]^+^.

***Bis*****((pivaloyloxy)methyl) chloro phosphate (5e)**. A solution of bis((pivaloyloxy)methyl) hydrogen phosphate **20** (400 mg, 1.22 mmol) and DMF (5 μL) in DCM (5 mL) was added into a solution of oxalyl chloride (580 μL, 6.12 mmol) in DCM (5 mL) at room temperature. The reaction was stirred for 2 h and closely monitored by ^31^P NMR. After the complete disappearance of the starting material was observed, the solvents and the by-products were removed under reduced pressure and the yellow oil was used for the next step without further purification. 415 mg, (98%). ^1^H NMR (500 MHz, CDCl_3_): *δ*=1.18 (18H, s, 2×C(C*H*_3_)_3_), 5.69 (2H, AB syst. *J*=3.1, 5.2 Hz, OC*H*_2_O), 5.72 ppm (2H, AB syst. *J*=5.2, 5.5 Hz, OC*H*_2_O). ^31^P NMR (202 MHz, CDCl_3_): *δ*=3.0 ppm.

**(5-(2,4-Dimethoxybenzyloxycarbamoyl)-2,2-dimethyl-1,3-dioxolan-4-yl)methyl bis((pivaloyloxy)methyl) phosphate (27)**. Triethylamine was added into a cooled (−78°C) solution of **4b** (82 mg, 0.24 mmol) in DCM (1 mL) followed by the slow addition of a solution of bis((pivaloyloxy)methyl) chloro phosphate **5e** (415 mg, 1.22 mmol) in dry DCM (1 mL) under argon atmosphere. The reaction was stirred 30 min at −78°C and then at room temperature for 1 hour. Monitored by ^31^P NMR and TLC (70% EtOAc in hexane). The reaction was quenched with a saturated solution of NH_4_Cl (2 mL), and the phases were separated. The organic portion was dried over Na_2_SO_4_, concentrated, and purified by chromatography eluting the column with a gradient of 40→60% EtOAc in hexane. The title compound was obtained in two different fractions, the first (10 mg) and the second slightly contaminated with some by-products (67 mg). *R*_f_ 0.32 (40% EtOAc/hexane). ^1^H NMR (500 MHz, CDCl_3_): *δ*=1.156 (9H, s, C(C*H*_3_)_3_), 1.160 (9H, s, C(C*H*_3_)_3_), 1.28 (3H, s, C(C*H*_3_)_2_), 1.40 (3H, s, C(C*H*_3_)_2_), 3.75 (3H, s, OC*H*_3_), 3.77 (3H, s, OC*H*_3_), 4.00–4.03 (1H, m, CHC*H*HOP), 4.38–4.40 (1H, m, CHCH*H*OP), 4.51–4.55 (2H, m, C*H*C*H*CH_2_), 4.84 (2H, AB syst. *J*=11.0 Hz, OC*H*_2_Ar), 5.57–5.60 (4H, m, 2×OC*H*_2_O), 6.40–6.42 (2H, m, Ar-H), 7.19–7.21 (1H, m, Ar-H), 8.82 ppm (1H, s, NH). ^13^C NMR (125 MHz, CDCl_3_): *δ*=24.6 (C(*C*H_3_)_2_), 26.7 (C(*C*H_3_)_2_), 26.8 (C(*C*H_3_)_3_), 38.7 (*C*(CH_3_)_3_), 55.4 (O*C*H_3_), 55.6 (O*C*H_3_), 66.7, 66.8 (CH*C*H_2_OP), 73.5 (O*C*H_2_Ar), 74.9 (*C*HCH), 75.8, 75.9 (CH*C*HCH_2_), 82.8, 82.9 (O*C*H_2_O), 98.6 (Ar-CH), 104.2 (Ar-CH), 110.8 (*C*(CH_3_)_2_), 132.9 (Ar-CH), 159.5 (Ar-C), 161.8 (Ar-C), 165.2 (HN*C*O), 176.7 ppm (*C*OC(CH_3_)_3_). ^31^P NMR (202 MHz, CDCl_3_): *δ*=−4.28 ppm. LRMS (ES+): *m*/*z* (%) 650.2 (40) [*M*+H]^+^, 651.2 (20) [*M*+H]^+^, 652.2 (5) [*M*+H]^+^, 667.3 (50) [*M*+NH_4_]^+^, 668.3 (20) [*M*+NH_4_]^+^, 669.3 (6) [*M*+NH_4_]^+^, 672.2 (100) [*M*+Na]^+^, 673.2 (35) [*M*+Na]^+^.

**(5-(hydroxycarbamoyl)-2,2-dimethyl-1,3-dioxolan-4-yl)methyl bis((pivaloyloxy)methyl) phosphate (3e)**. TFA (10 μL) was added into a solution of the second fraction of **27** (67 mg, 0.103 mmol ca). The reaction became deep purple and a white suspension was formed. The mixture was stirred for approximately 1 h, until the complete disappearance of the starting material was observed by TLC (60% EtOAc in hexane). The solvent was evaporated under reduced pressure, and the crude residue was taken in a mixture of DCM/Diethyl ether (1:1). The white precipitate was filtered off and washed with diethyl ether; the filtrate was concentrated (72 mg) and purified by column chromatography eluting with 60% EtOAc in DCM (20 mL), then with a gradient of 1→2% MeOH in DCM. The bis POM prodrug **3e** was obtained as yellowish oil (24 mg, 46%). *R*_f_ 0.19 (60% EtOAc in hexane). ^1^H NMR (500 MHz, CDCl_3_): *δ*=1.168 (9H, s, C(C*H*_3_)_3_), 1.169 (9H. S, C(C*H*_3_)_3_), 1.30 (3H, s, C(C*H*_3_)_2_), 1.48 (3H, s, C(C*H*_3_)_2_), 4.12–4.17 (1H, m, C*H*HOP), 4.22–4.26 (1H, m, CH*H*OP), 4.50–4.53 (1H. M, CHC*H*CH_2_), 4.67 (1H, d, *J*=7.6 Hz, C*H*CHCH_2_), 5.52–5.63 (4H, m, 2×OC*H*_2_O), 8.0 (0.6H, bs, OH), 9.02 ppm (1H, bs, NH). ^13^C NMR (125 MHz, CDCl_3_): *δ*=24.6 (C(*C*H_3_)_2_), 26.6 (C(*C*H_3_)_2_), 26.8 (C(*C*H_3_)_3_), 38.8 (*C*(CH_3_)_2_), 66.3, 66.4 (CH*C*H_2_OP), 74.8 (*C*HCH), 75.6, 75.7 (CH*C*HCH_2_O), 83.0, 83.1 (O*C*H_2_O), 110.8 (*C*(CH_3_)_2_), 165.5 (HN*C*O), 176.92, 176.94 ppm (*C*OC(CH_3_)_3_). ^31^P NMR (202 MHz, CDCl_3_): *δ*=−4.67 ppm. LRMS (ES+): *m*/*z* (%) 500.2 (100) [*M*+H]^+^, 501.2 (20) [*M*+H]^+^, 517.2 (30) [*M*+NH_4_]^+^, 522.2 (65) [*M*+Na]^+^, 523.2 (13) [*M*+Na]^+^. HRMS (ES+): required 522.1711 for C_19_H_34_NNaO_12_P, found 522.1696. LC-M: *R*_t_ 8.1 min; *m*/*z* 500; purity 90% by TIC trace.

**LC–MS stability experiments**. The phosphate buffer saline (PBS) purchased from Fluka was prepared dissolving 1 tablet in 200 mL of HPLC grade water (final pH 7.2).

The samples were prepared dissolving 0.5–1 mg of the prodrug in 1–0.5 mL of PBS buffer (to obtain a final concentration of 1 mgmL^−1^ ca), with the addition of DMSO in case of poor solubility in aqueous buffer. After dissolving the compounds in PBS a first analysis at t_0_ was carried out and the compounds were then stored in the incubator at 37°C. A 20 μL aliquot of the samples was taken hourly, diluted to 0.5 mL with a solution of 20% MeOH (LC–MS grade)/Water. The sample was then injected with 20 μL loop and eluted with the following gradient: 0→3′ 20% MeOH/H_2_O; 3→6′ 95% MeOH/H_2_O; 6→12.4′ 95% MeOH/H_2_O; 12.4→12.5 20% MeOH/H_2_O; 12.5→15′ 20% MeOH/H_2_O; using a Phenomenex Gemini 5u C18 110 A 50×3.0 mm column.

^**31**^**P NMR stability experiments**. Phosphate Buffer Saline was prepared dissolving half-tablet in 100 mL of deuterated water (GOSS Scientific >99.9 at.%), final pD 7.2.

The prodrugs (0.5–1 mg) were dissolved in 0.5 mL of deutared PBS buffer in a NMR tube and stored in the incubator at 37°C. NMR spectra were recorded a different times starting with t_0_ and then each hour until total decomposition was observed.

**In vitro Trypanocidal Assays (Bs427 strain)**. Bloodstream form trypanosomes of the Lister 427 strain were cultivated in HMI-9 medium supplemented with 10% fetal calf serum (Biosera, South America), penicillin/streptomycin, and β-mercaptoethanol. Cell growth was at 37°C in a humidified CO_2_ incubator. A modification of the Alamar Blue assay^24^ was used to determine IC_50_ values of the various compounds. Cells (2×10^5^) were added to 96-well plates with wells containing doubling dilution of each test compound (200 μL/well final volume, with the highest drug concentration of 100 μm) giving a final initial density of 1×10^6^ cells/mL. After 48 h, 20 μL of Alamar blue reagent (Bio-Source, Camarilo, CA) was added; 24 h later, the degree of blue-red color change was measured fluorimetrically (Perkin–Elmer LS55B). Dose-response curves were fitted using the Prism software (version 3.0, Graphpad). The 50% growth inhibitory concentrations (IC_50_) were determined from the sigmoidal inhibition profiles of the test compounds and are presented in Table 2. All experiments were performed in duplicate.

**Cytotoxicity to mammalian cells**. The cytotoxic effects of the compounds on mammalian cells were evaluated in human embryonic kidney cells (HEK) strain 293T just as described in the preceding section for trypanosome but with the following modifications:

1. Cells were cultured in Dulbecco’s modified Eagle’s medium (Sigma Chemical Co., St. Louis, MO), supplemented with 10% fetal bovine serum and 200 μm l-Glutamine and penicillin/streptomycin (Gibco).
2. Cells were seeded at 3×10^4^ per well and incubated 3 h to allow adhesion.
3. The highest drug concentration was 200 μm.
4. Alamar blue (20 μL) was added to the cells after 16 hours and the plates read after another 24 h.
